# Early time to recurrence predicts worse survival in patients with localized or regionally advanced cutaneous melanoma

**DOI:** 10.1111/dth.14981

**Published:** 2021-05-24

**Authors:** Chengcai Liang, Wanming Hu, Jingjing Li, Xiaoshi Zhang, Zhiwei Zhou, Yao Liang

**Affiliations:** ^1^ State Key Laboratory of Oncology in South China Collaborative Innovation Center for Cancer Medicine Guangzhou China; ^2^ Department of Gastric and Melanoma Surgery Sun Yat‐Sen University Cancer Center Guangzhou China; ^3^ Department of Pathology Sun Yat‐Sen University Cancer Center Guangzhou China; ^4^ Department of Medical Melanoma and Sarcoma Sun Yat‐sen University Cancer Center Guangzhou China

**Keywords:** cutaneous melanoma, overall survival, survival after recurrence, time to recurrence

## Abstract

To investigate the prognostic significance of time to recurrence (TTR) for overall survival (OS) and survival after recurrence (SAR) in patients with localized or regionally advanced cutaneous melanoma. A total of 731 cutaneous melanoma patients with an initial diagnosis of 8th American Joint Committee on Cancer (AJCC) clinical stage I‐III were included in this study. The prognostic factors associated with OS and SAR were estimated through Kaplan‐Meier and Cox regression analysis. Of the total cohort, 329 patients (45%) died, and 418 patients (57%) experienced recurrence. The median follow‐up and TTR were 55.6 months and 9.6 months, respectively. A total of 141 patients (19%) experienced recurrence in <6 months, and 277 patients (38%) experienced recurrence in ≥6 months. Patients with stage III and positive lymph node dissection (LND) were more common in the early TTR group than in the late TTR group. Both the OS and SAR rates at 5 years and 10 years in the early TTR group were significantly poorer than those in the late TTR group (*P* < .001 and *P* = .008, respectively). Furthermore, early TTR, along with truncal tumor, higher TNM stage and therapeutic variables (extended resection, LND and adjuvant therapy), were significant independent predictors of worse OS and SAR in multivariate analysis (all *P* < .05). Early TTR predicts worse survival and could be considered an independent prognostic factor for patients with localized or regionally advanced cutaneous melanoma. TTR should be evaluated in all patients with recurrence to guide post‐recurrence risk stratification and follow‐up schedules.

## 1 INTRODUCTION

Melanoma is becoming an increasingly prominent malignancy that has clear heterogeneity and ethnic variations worldwide. It is common in Western regions, such as Queensland and Australia, among people with light‐colored skin. However, this malignancy is relatively rare among Africans, Hispanics and Asians.^1^, ^2^, ^3^ As the most common subtype of melanoma, cutaneous melanoma usually occurs on the back, chest, abdomen and lower or upper extremity. Surgical resection of all lesions remains a curative treatment for patients with localized or regionally advanced cutaneous melanoma (ie, American Joint Committee on Cancer [AJCC] stage I, II and III), while metastatic melanoma relies on comprehensive treatments.^4^ Owing to oncological relapse, approximately 20% of patients with cutaneous melanoma will eventually develop metastatic disease and become incurable, with a 10‐year survival rate of less than 10%.^5^, ^6^ Thus, the early detection of disease recurrence is of clinical significance for this malignancy.

Currently, various prognostic factors have been proved to be associated with survival and recurrence in cutaneous melanoma, including tumor characteristics and pathological and therapeutic variables (such as extended resection of primary lesions, lymph node dissection (LND) and adjuvant therapy).^6^, ^7^, ^8^, ^9^ Among these potential prognostic factors, only a few publications have reported time to recurrence (TTR), and its prognostic influence remains controversial. However, in patients with cancers such as rectal cancer, renal cell carcinoma or gastric cancer, patients with early TTR were reported to have worse outcomes than those with late TTR.^10^, ^11^, ^12^ On the other hand, survival after recurrence (SAR) is an optimal study endpoint in malignancies with a high prevalence of recurrence, but the prognostic predictors of SAR in cutaneous melanoma have not been well discussed. Such knowledge is, however, valuable for assessing post‐recurrence risk stratification and guiding the appropriate follow‐up schedule.

Therefore, the aim of this study was to identify the prognostic significance of TTR as well as significant predictors of SAR in patients with localized or regionally advanced cutaneous melanoma.

## 2 METHODS

### 2.1 Study population

After the approval of our institutional review board, we reviewed 1530 initial patients with melanoma who underwent at least primary lesion resection with or without LND at our Cancer Center from January 1995 to December 2016. The flow of patient enrolment is shown in Figure S1. Those urological and gastrointestinal melanomas, recurrent and metastatic cutaneous melanomas, or lesions located in head or neck, were all excluded. Finally, 731 patients with an initial diagnosis of 8th AJCC stage I, II, or III cutaneous melanoma were enrolled into our study.

Among these patients, 418 patients (57%) were diagnosed with recurrence as a first event. TTR was calculated from the date of resection to the date of initial recurrence, then these recurrent patients were classified into two groups according to the duration of TTR. Patients who were diagnosed with TTR within 6 months (n = 141, 19%) were grouped into the “early TTR” group; patients who were diagnosed with TTR in no less than 6 months (n = 277, 38%) were included in the “late TTR” group.

### 2.2 Data collection and treatment

The clinical and pathological characteristics of all patients were collected from medical records. The results from sentinel lymph node biopsy (SLNB) were collected from clinical notes and pathological test reports. Tumor stage was assessed according to the AJCC 8th edition melanoma staging system.^5^

According to the published guidelines,^4^ extended excision was defined as the primary tumor resection with a safety margin of 1 cm for Breslow thickness below 2 mm and 2 cm for Breslow thickness above 2 mm. SLNB was indicated in patients with a tumor thickness of >1 mm or > 0.75 mm accompanied with additional risk factors such as mitotic rate or ulceration. Regional lymph node dissection (LND) was recommended for patients with positive SLNB or stage III disease diagnosed by physical examination, imaging or pathology.

Adjuvant therapy refers to those supplementary treatments except surgery, which mainly consists of adjuvant radiotherapy, high‐dose interferon α‐2b and cytotoxic drugs such as dacarbazine, temozolomide or other chemotherapy drugs. Generally, patients with high‐risk (stage IIB ~ IIIA) and very high‐risk (stage IIIB~IIIC) melanoma were recommended to receive adjuvant therapy. Adjuvant radiotherapy was only considered in cases of inadequate surgical resection or bulky disease.

### 2.3 Follow‐up

An independent follow‐up department in our hospital performed regular follow‐up. The latest follow‐up date of this study (October first, 2019) or death was considered as the final survival follow‐up time. Overall survival (OS) was defined as the time from primary tumor resection to any cause death or the last follow‐up. Recurrence‐free survival (RFS) was defined as the time from the resection to the first recurrence or the last follow‐up. SAR was calculated from the date of the first recurrence until the date of death or the last follow‐up. For recurrent patients, we evaluated the type of first recurrence, duration of TTR, presence of distant metastases (either as first or subsequent recurrence), and SAR. In accordance with that in previous studies,^7^, ^13^ the type of first recurrence was classified as local recurrence (within 5 cm of the primary scar), intralymphatic recurrence, regional lymph node recurrence and distant recurrence (distant organs or lymph nodes).

### 2.4 Statistical analysis

Comparisons of clinicopathological features between groups were performed using the chi‐square test, Fisher's exact test for categorical data, or the Student's *t* test for continuous variables. OS and SAR were estimated through **t**he Kaplan‐Meier method and the log‐rank test. Prognostic factors associated with OS, RFS and SAR were incorporated into univariate and multivariate analyses through a Cox proportional hazards models. A *P* value of <.05 was considered statistical significant. All statistical analyses were carried out using PASW version 18.0 statistical software (IBM Corp, Somers, New York).

## 3 RESULTS

The characteristics of the 731 patients are shown in Table 1. As depicted, the median age was 53 years (interquartile range [IQR]: 42 ~ 63 years). There were 375 male patients and 356 female patients with a male‐to‐female ratio of 1.05:1. The majority of patients were diagnosed with cutaneous melanoma on the lower extremity (526, 72%). Most patients had stage III disease (386, 52.8%), 90 patients (12.3%) had stage I disease, and 255 patients (34.9%) had stage II disease. Adjuvant therapy was administered to 407 patients (55.7%), including five patients (0.7%) with adjuvant radiotherapy, 382 patients (52.3%) with adjuvant drug therapy and 20 patients (2.7%) with both treatments.

**TABLE 1:** Characteristics of the 731 total patients

Variables	Total (n = 731)
Age median, months, (IQR)	53 (42‐63)
OS median, months, (IQR)	55.6 (33.9‐94.2)
SAR median, months, (IQR)	25.5 (13.6‐50)
RFS median, months, (IQR)	28.9 (8.1‐70.6)
TTR median, months, (IQR)	9.6 (4.8‐20.7)
Sex, no. (%)	
Female	356 (48.7%)
Male	375 (51.3%)
Size, no. (%)	
≤5 cm	561 (76.7%)
>5 cm	72 (9.8%)
Not available	98 (13.5%)
Topography, n (%)	
Lower extremity	526 (72%)
Upper extremity	106 (14.5%)
Trunk	99 (13.5%)
Physical stimulation, no. (%)	
No	529 (72.4%)
Yes	202 (27.6%)
Extended resection, no. (%)	
No	350 (47.9%)
Yes	381 (52.1%)
SLNB, no. (%)	
Negative	158 (21.6%)
Positive	69 (9.4%)
Not done	504 (69%)
LND, no. (%)	
Negative	52 (7.1%)
Positive	203 (27.8%)
Not done	476 (65.1%)
8th AJCC stage, no. (%)	
I	90 (12.3%)
II	255 (34.9%)
III	386 (52.8%)
Surgical margin, no. (%)	
Negative	476 (65.1%)
Positive	33 (4.5%)
Not available	222 (30.4%)
Adjuvant therapy, no. (%)	
No	324 (44.3%)
Yes	407 (55.7%)

Table 2 demonstrated the clinicopathological features of recurrent patients. As shown, compared with the late TTR group, the early TTR group had significantly shorter median OS, SAR and TTR times (all *P* < .05). In addition, significantly more patients with positive LND and stage III disease were found in the early TTR group, whereas the two groups were relatively well balanced with regard to age, sex, size, tumor site, physical stimulation, tumor thickness, ulceration, extended resection, surgical margin and adjuvant therapy.

**TABLE 2:** Characteristics of the 418 recurrent patients

Variables	Late TTR (n = 277, 38%)	Early TTR (n = 141, 19%)	*P* value
Age median, months, (IQR)	53 (42‐61)	53 (42‐62)	.885
OS median, months, (IQR)	52.5 (32.2‐83.9)	26.9 (17.2‐46.7)	**<.001**
SAR median, months, (IQR)	28 (13.6‐55)	23 (13.7‐42.9)	**.024**
TTR median, months, (IQR)	15.7 (9.8‐28.8)	4 (2.9‐4.8)	**<.001**
Sex, no. (%)			.848
Female	123 (44.4%)	64 (45.4%)	
Male	154 (55.6%)	77 (54.6%)	
Size, no. (%)			.621
≤5 cm	191 (69%)	91 (64.5%)	
>5 cm	37 (13.3%)	20 (14.2%)	
Not available	49 (17.7%)	30 (21.3%)	
Topography, n (%)			.734
Lower extremity	187 (67.5%)	100 (70.9%)	
Upper extremity	41 (14.8%)	20 (14.2%)	
Trunk	49 (17.7%)	21 (14.9%)	
Physical stimulation, no. (%)			.371
No	204 (73.6%)	98 (69.5%)	
Yes	73 (26.4%)	43 (30.5%)	
Extended resection, no. (%)			.94
No	162 (58.5%)	83 (58.9%)	
Yes	115 (41.5%)	58 (41.1%)	
SLNB, no. (%)			.063
Negative	31 (11.2%)	14 (9.9%)	
Positive	21 (7.6%)	21 (14.9%)	
Not done	225 (81.2%)	106 (75.2%)	
LND, no. (%)			**.005** ^a^
Negative	23 (8.3%)	3 (2.1%)	
Positive	69 (24.9%)	51 (36.2%)	
Not done	185 (66.8%)	87 (61.7%)	
8th AJCC stage, no. (%)			**.023**
I	7 (2.5%)	5 (3.6%)	
II	81 (29.3%)	24 (17%)	
III	189 (68.2%)	112 (79.4%)	
Surgical margin, no. (%)			.475
Negative	149 (53.8%)	77 (54.6%)	
Positive	14 (5%)	11 (7.8%)	
Not available	114 (41.2%)	53 (37.6%)	
Adjuvant therapy, no. (%)			.34
No	81 (29.2%)	35 (24.8%)	
Yes	196 (70.8%)	106 (75.2%)	

*Note*: The bold values are statistical significance values with *p* < 0.05.

^a^ Fisher's exact tests.

During a median follow‐up time of 55.6 months (IQR: 33.9 ~ 94.2 months), 329 patients (45%) died, and 418 patients (57%) experienced recurrence. Table S1 shows the patterns of first recurrence and final distant metastasis. As depicted, regional lymph node recurrence was the most common type of first recurrence (196, 46.9%). Eventually, of the total cohort, 282 (38.6%) patients developed distant metastasis. Significantly more patients with early TTR developed distant metastasis than those with late TTR (71.6% vs 65.3%, *P* < .001). The recurrence rates of stages I, II, and III melanoma were 13.3%, 41.2% and 78%, respectively. Furthermore, Table 3 shows the univariate and multivariate Cox regression analyses for predicting RFS. As shown in the multivariate Cox regression, large size (hazard ratio [HR] = 1.653, *P* < .001), higher TNM stage (stage II vs I: HR = 3.418, *P* < .001; stage III vs I: HR = 6.471, *P* < .001), positive surgical margin (HR = 2.654, *P* < .001) and adjuvant therapy (HR = 1.919, *P* < .001) were significantly associated with poor RFS.

**TABLE 3:** Univariate and multivariate analysis predicting RFS in 731 melanoma patients

Variables	RFS Univariate analysis			RFS Multivariate analysis		
	HR	95% CI	*P* value	HR	95% CI	*P* value
Age continuous	0.998	0.992 ~ 1.004	.532			
Sex (male vs female)	1.275	1.058 ~ 1.537	**.011**	1.215	0.929 ~ 1.362	0.226
Size (>5 cm vs ≤5 cm)	2.105	1.591 ~ 2.785	**<.001**	1.653	1.248 ~ 2.188	**<.001**
Topography			**.026**	1.076		.682
Upper extremity vs Lower extremity	1.095	0.838 ~ 1.431	.506	1.008	0.769 ~ 1.322	.953
Trunk vs Lower extremity	1.438	1.116 ~ 1.854	**.005**	1.124	0.862 ~ 1.466	.387
TNM			**<.001**			**<.001**
II vs I	4.043	2.281 ~ 7.168	**<.001**	3.418	1.923 ~ 6.073	**<.001**
III vs I	10.256	5.88 ~ 17.887	**<.001**	6.471	3.668 ~ 11.415	**<.001**
Physical stimulation (Yes vs No)	1.065	0.865 ~ 1.311	.551			
Extended resection (No vs Yes)	1.754	1.453 ~ 2.118	**<.001**	1.013	0.803 ~ 1.278	.913
Surgical margin			**<.001**			**<.001**
Positive vs Negative	3.001	2.047 ~ 4.4	**<.001**	2.654	1.808 ~ 3.895	**<.001**
Not available vs Negative	2.08	1.712 ~ 2.527	**<.001**	1.487	1.212 ~ 1.825	**<.001**
LND			.281			
Positive vs Negative	0.98	0.68 ~ 1.413	.915			
Not done vs Negative	1.179	0.957 ~ 1.452	.122			
Adjuvant therapy (Yes vs No)	2.628	2.146 ~ 3.217	**<.001**	1.919	1.557 ~ 1.366	**<.001**

*Note*: The bold values are statistical significance values with *p* < 0.05.

**FIGURE 1:**
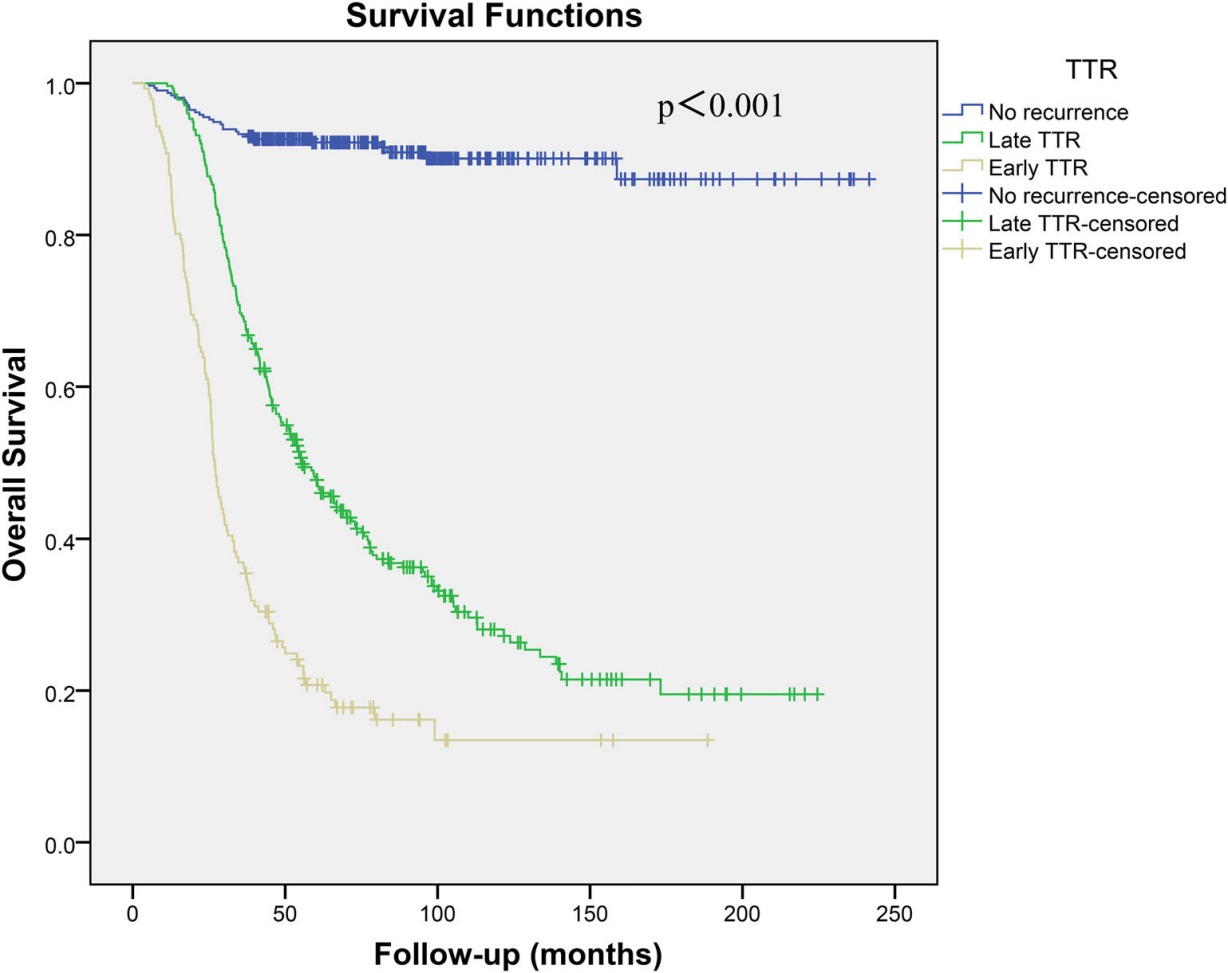
Kaplan–Meier curves depicting overall survival stratified by time to recurrence (TTR) in 731 melanoma patients

**FIGURE 2:**
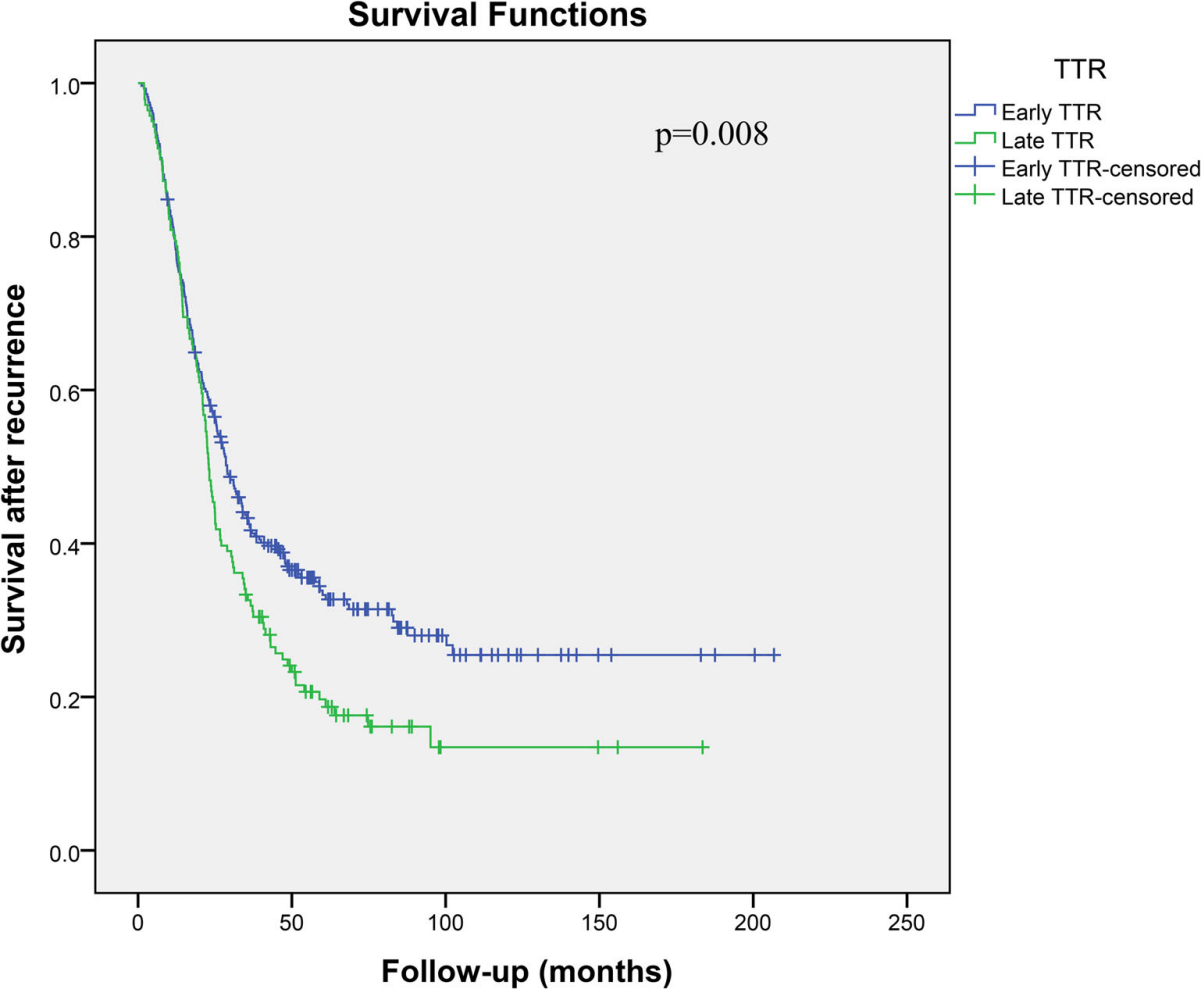
Kaplan–Meier curves depicting survival after recurrence stratified by time to recurrence (TTR) in 418 recurrent melanoma patients

The median TTR of recurrent patients was 9.6 months (IQR: 4.8 ~ 20.7 months). Figures 1, 2 display OS and SAR curves according to TTR, respectively. The 5‐year and 10‐year OS rates of patients with early TTR were significantly poorer than those of patients with late TTR and those of patients without recurrence (20.7% and 13.5% vs 47.7% and 28.1% vs 92.2% and 90.1%, respectively; *P* < .001). Additionally, the 5‐year and 10‐year SAR rates of patients with early TTR were significantly poorer than those of patients with late TTR (19.7% and 13.4% vs 33.3% and 25.5%, respectively; *P* = .008).

Furthermore, Tables 4, 5 show the prognostic factors associated with OS and SAR. As shown, truncal tumor (HR = 1.462, *P* = .014), tumor thickness (>4 vs ≤2 mm: HR = 1.812, *P* = .014), higher TNM stage (stage II vs I: HR = 6.26, *P* = .012; stage III vs I: HR = 11.119, *P* = .001), no extended resection (HR = 1.644, *P* < .001), positive LND (HR = 2.454, *P* < .001), adjuvant therapy (HR = 1.309, *P* = .037) and early TTR (late TTR vs no recurrence: HR = 6.579, *P* < .001; early TTR vs no recurrence: HR = 15.678, *P* < .001) were significantly associated with worse OS (Table 4). Additionally, truncal tumor (HR = 1.461, *P* = .017), higher TNM stage (stage II vs I: HR = 7.984, *P* = .04; stage III vs I: HR = 15.309, *P* = .007), no extended resection (HR = 1.361, *P* = .03), positive LND (HR = 1.879, *P* = 0.019), adjuvant therapy (HR = 1.444, *P* = .009) and early TTR (early TTR vs late TTR: HR = 1.348, *P* = .013) were significantly associated with worse SAR (Table 5).

**TABLE 4:** Univariate and multivariate analysis predicting OS in 731 melanoma patients

Variables	OS Univariate analysis			OS Multivariate analysis		
	HR	95% CI	*P* value	HR	95% CI	*P* value
Age continuous	0.998	0.991 ~ 1.006	.625			
Sex (male vs female)	1.323	1.063 ~ 1.646	**.012**	1.033	0.825 ~ 1.294	.776
Size (>5 cm vs ≤5 cm)	1.924	1.395 ~ 2.653	**<.001**	1.340	1.007 ~ 1.784	**.045**
Topography			**.006**			.061
Upper extremity vs Lower extremity	1.41	1.049 ~ 1.896	**.023**	1.103	0.818 ~ 1.488	.521
Trunk vs Lower extremity	1.504	1.123 ~ 2.014	**.006**	1.453	1.076 ~ 1.961	**.015**
TNM			**<.001**			**<.001**
II vs I	14.77	3.621 ~ 60.238	**<.001**	7.865	1.916 ~ 32.283	**.004**
III vs I	45.981	11.434 ~ 184.906	**<.001**	13.757	3.356 ~ 56.386	**<.001**
Physical stimulation (Yes vs No)	1.054	0.827 ~ 1.343	.669			
Extended resection (No vs Yes)	1.405	1.129 ~ 1.748	**.002**	1.562	1.196 ~ 2.04	**.001**
Surgical margin			**<.001**			**.003**
Positive vs Negative	2.122	1.332 ~ 3.379	**.002**	1.204	0.740 ~ 1.96	.454
Not available vs Negative	2.283	1.826 ~ 2.854	**<.001**	1.577	1.21 ~ 2.055	**<.001**
LND			**.006**			**.002**
Negative vs Not done	1.236	0.803 ~ 1.903	.336	1.290	0.988 ~ 1.686	.061
Positive vs Not done	1.478	1.166 ~ 1.874	**.001**	2.243	1.413 ~ 3.562	**.001**
Adjuvant therapy (Yes vs No)	2.758	2.161 ~ 3.519	**<.001**	1.306	1.014 ~ 1.68	**.038**
TTR			**<.001**			**<.001**
Late TTR vs No recurrence	9.692	6.511 ~ 14.428	**<.001**	6.376	4.18 ~ 9.728	**<.001**
Early TTR vs No recurrence	21.601	14.229 ~ 32.791	**<.001**	15.226	9.741 ~ 23.8	**<.001**

*Note*: The bold values are statistical significance values with *p* < 0.05.

**TABLE 5:** Univariate and multivariate analysis predicting SAR in 418 recurrent melanoma patients

Variables	SAR Univariate analysis			SAR Multivariate analysis		
	HR	95% CI	*P* value	HR	95% CI	*P* value
Age continuous	0.995	0.987 ~ 1.003	.211			
Sex (male vs female)	1.129	0.899 ~ 1.419	.297			
Size (>5 cm vs ≤5 cm)	1.176	0.845 ~ 1.637	.336			
Topography			**.019**			.064
Upper extremity vs Lower extremity	1.217	0.898 ~ 1.648	.206	1.157	0.848 ~ 1.578	.358
Trunk vs Lower extremity	1.563	1.147 ~ 2.128	**.005**	1.461	1.069 ~ 1.998	**.017**
TNM			**<.001**			**<.001**
II vs I	8.931	1.235 ~ 64.574	**.03**	7.984	1.102 ~ 57.852	**.04**
III vs I	18.464	2.588 ~ 131.707	**.004**	15.309	2.135 ~ 109.773	**.007**
Physical stimulation (Yes vs No)	0.939	0.73 ~ 1.209	.626			
Extended resection (No vs Yes)	1.271	1.01 ~ 1.598	**.041**	1.361	1.03 ~ 1.8	**.03**
Surgical margin			**.033**			.067
Positive vs Negative	0.725	0.426 ~ 1.233	.235	0.861	0.498 ~ 1.491	.594
Not available vs Negative	1.263	1.001 ~ 1.593	**.049**	1.315	1.007 ~ 1.718	**.044**
LND			**<.001**			**.05**
Negative vs Not done	1.294	0.787 ~ 2.128	.309	1.205	0.914 ~ 1.588	.186
Positive vs Not done	1.72	1.342 ~ 2.205	**<.001**	1.879	1.112 ~ 3.176	**.019**
Adjuvant therapy (Yes vs No)	1.741	1.327 ~ 2.285	**<.001**	1.444	1.095 ~ 1.905	**.009**
TTR (early TTR vs late TTR)	1.369	1.084 ~ 1.729	**.008**	1.348	1.064 ~ 1.708	**.013**

## 4 DISCUSSION

In this cohort, which included 731 patients with localized or regionally advanced cutaneous melanoma, the overall prognosis was suboptimal, with 418 (57%) patients experiencing recurrence and 329 (45%) patients dying. Lymph node recurrence (196, 46.9%) ranked among the most common types of recurrence. Most importantly, we investigated significant predictors of SAR and the prognostic influence of TTR. The results revealed that early TTR predicts worse survival and could be considered an independent prognostic factor for patients with localized or regionally advanced cutaneous melanoma. Thus, TTR should be evaluated in all patients with recurrence to guide post‐recurrence risk stratification and follow‐up schedules.

As mentioned above, we detected significantly more patients with positive LND and stage III disease in the early TTR group, whereas the two groups were relatively well balanced with regard to age, sex, size, tumor site, physical stimulation, tumor thickness, ulceration, extended resection, surgical margin and adjuvant therapy. Lymph node status and tumor stage are reflections of tumor's invasiveness, thus these results suggested that LND and tumor invasiveness might play the most crucial roles in the duration of TTR.

The duration of TTR is a valuable variable for assessing the risk of disease recurrence and determining the appropriate follow‐up schedule for many cancers. It has been reported that patients with late recurrence had better survival than those with early recurrence among those with rectal cancer, renal cell carcinoma and gastric cancer.^10^, ^11^, ^12^ In cutaneous melanoma, despite several publications investigating the prognostic impact of TTR, the results remain controversial. The studies from Peters et al.^14^ and Soong et al.^15^ did not find that TTR had any influence on survival, while the study from Dong et al.^16^ showed significantly poorer outcomes for patients with early TTR than for those with late TTR (*P* = .0098). Another study^17^ revealed that extraordinarily short (<1 year) and long (>10 years) times to the first recurrence had a negative effect on survival in the univariate analysis, but this finding was not confirmed in the multivariate analysis. In this study, we confirmed that TTR was one of the most influential prognostic factors for both OS and SAR in the multivariate analysis.

SAR is an optimal study endpoint for cutaneous melanoma, which is characterized by a relatively high rate of recurrence after initial treatment. However, compared with OS, there is much less work exclusively assessing the predictors of SAR. In our study, along with TTR, tumor site, TNM stage and therapeutic variables (extended resection, LND and adjuvant therapy) remained the most crucial predictors of both OS and SAR. This result corroborated with those of previous reports,^8^, ^9^, ^18^, ^19^ which indicated that the biological invasiveness of the primary tumor might be maintained in the subsequent local recurrence and confirmed the prognostic importance of the radicality of initial treatment.

Long‐term follow‐up is inevitable for the analysis of time to first recurrence and SAR. The median follow‐up time of our study was 55.6 months (IQR: 33.9 ~ 94.2 months). Usually, the recommended follow‐up for melanoma is 5 to 10 years. In addition, the median TTR of recurrent patients was 9.6 months (IQR: 4.8 ~ 20.7 months), which was shorter than that in other reports^17^ (median 24 months). The difference could be attributed to the higher proportion of patients with advanced stages in this cohort. Such detailed knowledge about TTR and SAR could be valuable in guiding an appropriate follow‐up program and improve patients' compliance during the follow‐up.

Our study was based on a large cohort of cutaneous melanoma patients from a high‐level Chinese cancer center. Though there have been numerous studies focused on melanoma, the findings of melanoma in Chinese patients are still limited. There are even less data concerning predictors of SAR in the Chinese melanoma population. Thus, this study provided additional prognostic information and could promote a better understanding of this malignancy in the Chinese population. Moreover, according to previous studies,^20^, ^21^, ^22^ substantial differences in clinical and pathological features of melanoma have been observed between Chinese patients and Caucasian patients. First, the incidence of malignant melanoma in China is much lower than that in European and American countries. However, the growth rate of new cases in China is rapid, with more than 20 000 new cases and approximately a growth rate of 3% to 5% per year.^20^, ^21^, ^22^ Second, the majority of Chinese patients are diagnosed with locally advanced stages. As an example, in our study, 386 patients (52.8%) had stage III disease, and 255 patients (34.9%) had stage II disease. In contrast, stage I patients accounted for 82% ~ 85% of Caucasian patients.^22^ Third, more than 80% of skin melanomas in Caucasian patients are located on the head and face, while the most frequent anatomic sites in Chinese patients are the feet and hands.^23^

The survival and recurrence of patients with cutaneous melanoma after surgery in Western counties has been extensively discussed for a long time. Regarding survival, patients in Western counties have a rather favorable prognosis, with 5‐year OS rates ranging from ~95% to 100% for patients with stage I, from ~65 to 93% for patients with stage II and from ~4%1 to 71% for patients with stage III. Recurrence rates also significantly vary among different tumor stages, with 5‐year RFS rates of approximately 56% in stage II patients and 28 to 44% in stage III patients.^6^, ^18^, ^24^ Consistent with these reports, in this study, the 5‐year stage‐specific OS rates were 97.8% for stage I, 76.1% for stage II and 42.7% for stage III; the 5‐year stage‐specific RFS rates were 53.4% for stage II and 23.5% for stage III. However, in a study from Guo et al.,^25^ which enrolled a total of 522 melanoma cases, and the results demonstrated that the 5‐year OS rates of patients with stage I, II, and III disease were 94.1%, 44.0% and 38.4%, respectively. Their results indicated that the prognosis of Chinese patients with malignant melanoma was suboptimal. However, it is noteworthy that their cohort was mixed with mucosal melanoma and other subtypes. Overall, there is large variability in survival and recurrence among different counties and regions.

In addition, we investigated risk factors for RFS. The results showed that large size, positive surgical margin and higher TNM stage were significantly associated with poor RFS. These findings were consistent with those of previous studies.^7^ It is noteworthy that adjuvant therapy was significantly associated with worse survival and higher recurrence. This phenomenon might be attributed to the higher proportion of patients with advanced stages and unfavorable tumor characteristics in the adjuvant therapy group. Moreover, variables such as sex, tumor site, tumor thickness, ulceration, SLNB and extended resection were significantly associated with RFS in the univariate analysis. Therefore, male patients with truncal tumors without extended resection should probably be given more attention in long‐term follow‐up.

Finally, there are some limitations to be noted. First, this was a retrospective study with the accompanying inherent sources of bias. Second, the enrolled patients received treatment over a spanning period of over 20 years, during which great progress in treating melanoma had occurred. Third, all patients enrolled in this study were selected from one hospital, which means our conclusions should be verified in a larger population from multiple centers. In addition, some clinicopathological variables were not incorporated into the analysis, such as mitotic rate and histologic subtypes. These data were not obligatory in previous pathological reports, consequently leading to missing data in many patients.

## 5 CONCLUSION

Early TTR predicts worse survival and could be considered an independent prognostic factor for patients with localized or regionally advanced cutaneous melanoma. TTR should be evaluated in all patients with recurrence to guide post‐recurrence risk stratification and follow‐up schedules.

## CONFLICTS OF INTEREST

The authors have no conflict of interest to disclose.

## AUTHORS' CONTRIBUTIONS

Protocol development: Chengcai Liang, Yao Liang; Data collection and analysis: Wanming Hu, Jingjing Li; Manuscript writing: Chengcai Liang, Yao Liang; Manuscript editing: Xiaoshi Zhang, Zhiwei Zhou. All authors agreed to be responsible for all aspects of the study. All authors read and approved the final manuscript.

## ETHICS STATEMENT

This study was also approved by the independent Institute Research Ethics Committee at the Sun Yat‐sen University Cancer Center. We conducted this retrospective research according to the principles expressed in the Declaration of Helsinki.

## Supporting information

**Figure S1** The flow of patient enrolment.

**Table S1** Type of first recurrence and final metastasis in 418 recurrent patients

## Data Availability

The authenticity of this article was validated by uploading the key raw data to the Research Data Deposit (RDD) public platform (www.researchdata.org.cn) with the approval RDD number of RDDA2020001487.
