# Supplementary figures and images for: Early time to recurrence predicts worse survival in patients with localized or regionally advanced cutaneous melanoma
Source: Dermatol Ther. 2021 May 24;34(4):e14981. doi: 10.1111/dth.14981 (PMC8459230; doi:10.1111/dth.14981)

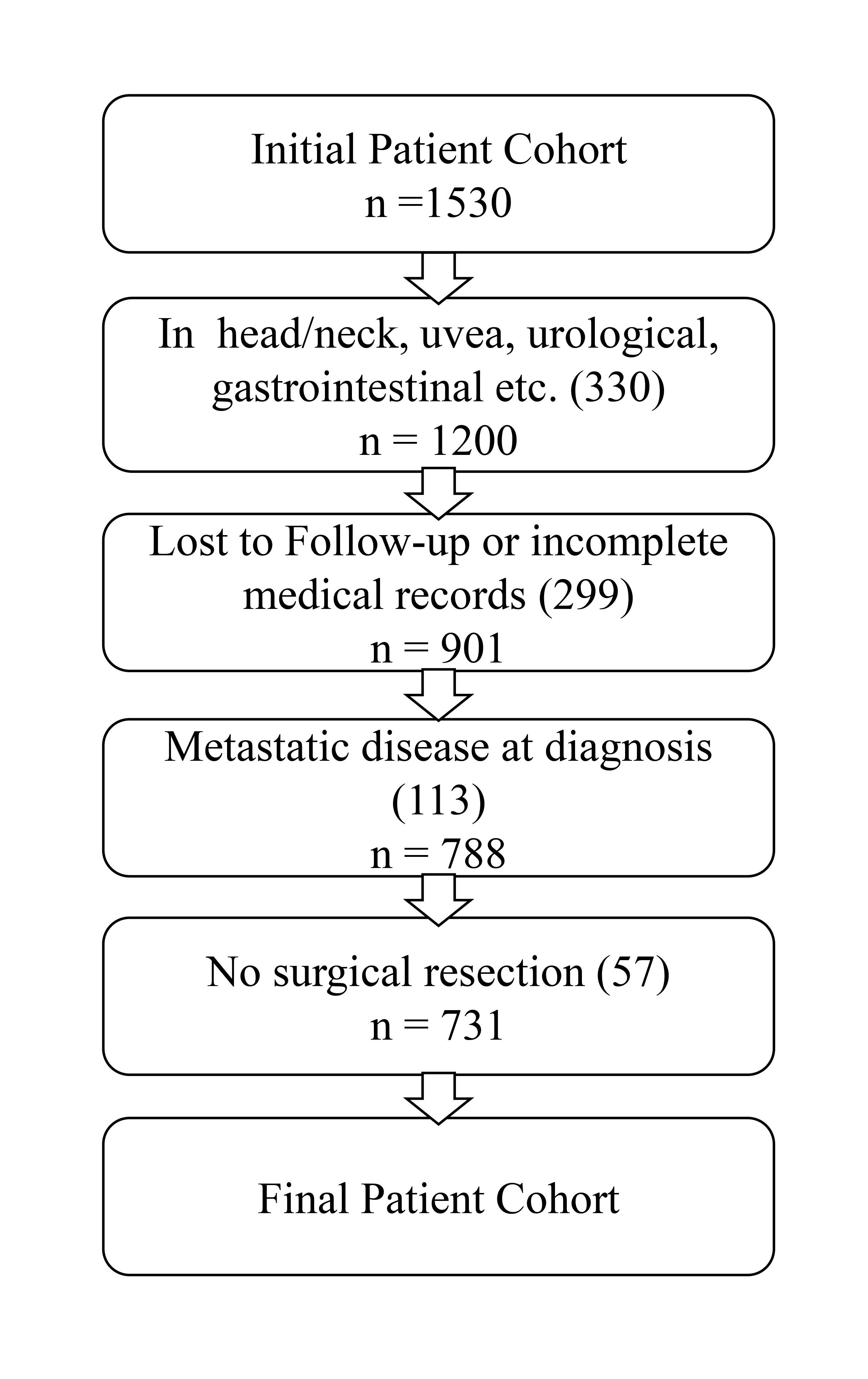

Supplement: Supplementary file 1 — Figure S1 The flow of patient enrolment. [file DTH-34-e14981-s002.tif]
