# Supplementary material for: Early time to recurrence predicts worse survival in patients with localized or regionally advanced cutaneous melanoma
Source: Dermatol Ther. 2021 May 24;34(4):e14981. doi: 10.1111/dth.14981 (PMC8459230; doi:10.1111/dth.14981)
Supplement: Supplementary file 2 — Table S1 Type of first recurrence and final metastasis in 418 recurrent patients [file DTH-34-e14981-s001.docx]

| Supplementary Table 1 Type of first recurrence and final metastasis in 418 recurrent patients | | | | |
| --- | --- | --- | --- | --- |
| Type of first recurrence, n (%) | Total (n=418) | Late TTR (n=277, 38%) | early TTR (n=141, 19%) | p-value |
| Local recurrence | 126 (30%) | 80 (28.9%) | 46 (32.6%) | 0.43 |
| Intralymphatic recurrence | 65 (15.6%) | 42 (15.2%) | 23 (16.3%) | 0.759 |
| Regional lymph node recurrence | 196 (46.9%) | 132 (47.7%) | 64 (45.4%) | 0.661 |
| Distant recurrence | 119 (28.5%) | 78 (28.2%) | 41 (29.1%) | 0.844 |
| Final distant metastasis, n (%) |  |  |  | **<0.001** |
| No | 136 (32.5%) | 96 (34.7%) | 40 (28.4%) |  |
| Yes | 282 (67.5%) | 181 (65.3%) | 101 (71.6%) |  |
